# Risk assessment in patients with symptomatic and asymptomatic pre-excitation

**DOI:** 10.1093/europace/euae036

**Published:** 2024-02-15

**Authors:** Anette Jemtrén, Serkan Saygi, Finn Åkerström, Fahd Asaad, Tara Bourke, Frieder Braunschweig, Carina Carnlöf, Nikola Drca, Per Insulander, Göran Kennebäck, Astrid Paul Nordin, Bita Sadigh, Anette Rickenlund, Ott Saluveer, Jonas Schwieler, Emma Svennberg, Jari Tapanainen, Yusuf Turkmen, Hamid Bastani, Mats Jensen-Urstad

**Affiliations:** Heart and Lung Disease Unit, Department of Medicine Huddinge, Karolinska Institutet, Stockholm, Sweden; Department of Cardiology, Heart and Vascular Centre, Karolinska University Hospital Huddinge, Department of Medicine Huddinge, Karolinska Institutet, Hälsovägen, 141 86 Stockholm, Sweden; Department of Cardiology, Heart and Vascular Centre, Karolinska University Hospital Huddinge, Department of Medicine Huddinge, Karolinska Institutet, Hälsovägen, 141 86 Stockholm, Sweden; Department of Cardiology, Heart and Vascular Centre, Karolinska University Hospital Huddinge, Department of Medicine Huddinge, Karolinska Institutet, Hälsovägen, 141 86 Stockholm, Sweden; Department of Cardiology, Heart and Vascular Centre, Karolinska University Hospital Huddinge, Department of Medicine Huddinge, Karolinska Institutet, Hälsovägen, 141 86 Stockholm, Sweden; Department of Cardiology, Heart and Vascular Centre, Karolinska University Hospital Huddinge, Department of Medicine Huddinge, Karolinska Institutet, Hälsovägen, 141 86 Stockholm, Sweden; Department of Cardiology, Heart and Vascular Centre, Karolinska University Hospital Huddinge, Department of Medicine Huddinge, Karolinska Institutet, Hälsovägen, 141 86 Stockholm, Sweden; Department of Cardiology, Heart and Vascular Centre, Karolinska University Hospital Huddinge, Department of Medicine Huddinge, Karolinska Institutet, Hälsovägen, 141 86 Stockholm, Sweden; Department of Cardiology, Heart and Vascular Centre, Karolinska University Hospital Huddinge, Department of Medicine Huddinge, Karolinska Institutet, Hälsovägen, 141 86 Stockholm, Sweden; Department of Cardiology, Heart and Vascular Centre, Karolinska University Hospital Huddinge, Department of Medicine Huddinge, Karolinska Institutet, Hälsovägen, 141 86 Stockholm, Sweden; Department of Cardiology, Heart and Vascular Centre, Karolinska University Hospital Huddinge, Department of Medicine Huddinge, Karolinska Institutet, Hälsovägen, 141 86 Stockholm, Sweden; Department of Cardiology, Heart and Vascular Centre, Karolinska University Hospital Huddinge, Department of Medicine Huddinge, Karolinska Institutet, Hälsovägen, 141 86 Stockholm, Sweden; Department of Clinical Physiology, Karolinska University Hospital, Stockholm, Sweden; Department of Cardiology, Heart and Vascular Centre, Karolinska University Hospital Huddinge, Department of Medicine Huddinge, Karolinska Institutet, Hälsovägen, 141 86 Stockholm, Sweden; Department of Cardiology, Heart and Vascular Centre, Karolinska University Hospital Huddinge, Department of Medicine Huddinge, Karolinska Institutet, Hälsovägen, 141 86 Stockholm, Sweden; Department of Cardiology, Heart and Vascular Centre, Karolinska University Hospital Huddinge, Department of Medicine Huddinge, Karolinska Institutet, Hälsovägen, 141 86 Stockholm, Sweden; Department of Clinical Sciences, Danderyd Hospital Division of Cardiovascular Medicine, Karolinska Institutet, Stockholm, Sweden; Department of Cardiology, Heart and Vascular Centre, Karolinska University Hospital Huddinge, Department of Medicine Huddinge, Karolinska Institutet, Hälsovägen, 141 86 Stockholm, Sweden; Department of Cardiology, Heart and Vascular Centre, Karolinska University Hospital Huddinge, Department of Medicine Huddinge, Karolinska Institutet, Hälsovägen, 141 86 Stockholm, Sweden; Department of Cardiology, Heart and Vascular Centre, Karolinska University Hospital Huddinge, Department of Medicine Huddinge, Karolinska Institutet, Hälsovägen, 141 86 Stockholm, Sweden

**Keywords:** Wolff–Parkinson–White syndrome, Sudden death, Exercise stress test, Electrophysiological study

## Abstract

**Aims:**

Controversy remains as to whether the exercise stress test (EST) is sufficient for risk evaluation in patients with pre-excitation. This study aims to clarify the usefulness of EST in risk stratification in both asymptomatic and symptomatic patients presenting with pre-excitation.

**Methods and results:**

This prospective study includes consecutive asymptomatic and symptomatic patients with pre-excitation referred for risk assessment. All participants performed an incremental EST (bicycle) prior to an electrophysiology study (EPS). Primary data from the EST included loss of pre-excitation during exercise, and primary data from the EPS included the measurement of accessory pathway effective refractory period (APERP), shortest pre-excited RR interval (SPERRI), and inducible arrhythmia with the use of a beta-adrenergic receptor agonist if deemed necessary. One hundred and sixty-four patients (59 asymptomatic, 105 symptomatic) completed an EST and EPS. Forty-five patients (27%) demonstrated low-risk findings on EST, of which 19 were asymptomatic and 26 were symptomatic. Six patients with low-risk EST findings had SPERRI/APERP ≤ 250 ms at EPS, and two of them were asymptomatic. The sensitivity, specificity, positive predictive value, negative predictive value (NPV), and accuracy of low-risk EST for excluding patients with SPERRI/APERP ≤ 250 ms were 40, 91, 87, 51, and 60%, respectively. The number of patients with inducible arrhythmia at EPS was similar in the asymptomatic (36, 69%) and symptomatic (73, 61%) groups.

**Conclusion:**

Sudden loss of pre-excitation during EST has a low NPV in excluding high-risk APs. The EPS with the use of isoproterenol should be considered to accurately assess the risk of patients with pre-excitation regardless of symptoms (ClinicalTrials.gov Identifier: NCT03301935).

## Graphical Abstract

**Graphical Abstract:**
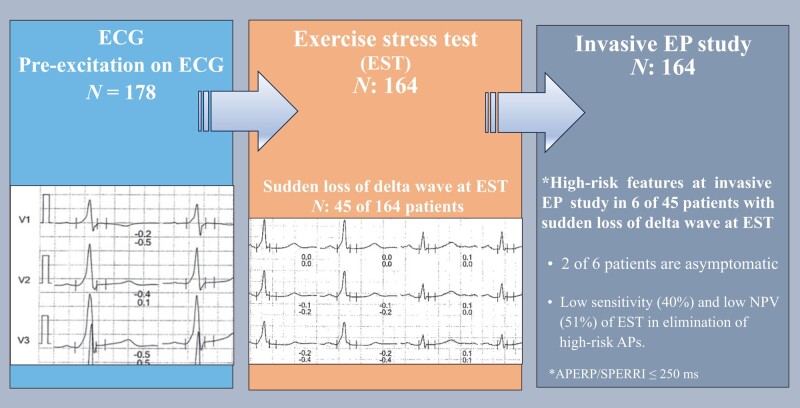

**What’s new?**

- Sudden loss of pre-excitation during exercise stress test has a low sensitivity and low negative predictive value to rule out potentially dangerous accessory pathways.
- Invasive electrophysiological study with the use of isoproterenol should be considered for accurate risk assessment in patients with pre-excitation regardless of symptoms.

## Introduction

Ventricular pre-excitation affects about 0.1–0.3% of the general population.^1–3^ When pre-excitation is accompanied by symptoms such as syncope or palpitations, the diagnosis of Wolff–Parkinson–White (WPW) syndrome is established.^4^ Patients with the WPW syndrome have an increased mortality rate with an incidence of sudden death of about 0.15% per year and may approach 3–4% over a lifetime.^2^ Risk stratification of symptomatic patients by performing an electrophysiological study (EPS) and catheter ablation of the accessory pathway (AP) are recommended in current guidelines (Class I).^5^ Variables on the EPS that identify patients with a high-risk AP include shortest pre-excited RR interval (SPERRI) ≤ 250 ms, accessory pathway effective refractory period (APERP) ≤ 250 ms, multiple APs, and an inducible AP-mediated tachycardia at baseline or during isoprenaline infusion.^6^

Risk stratification in asymptomatic individuals presenting with pre-excitation has been debated for years, and management of such patients remains controversial. According to present guidelines, an EPS for risk evaluation may be considered in asymptomatic pre-excitation.^7^ However, performance of an EPS, with the use of isoproterenol, is recommended in individuals with asymptomatic pre-excitation who have high-risk occupations/hobbies and those who participate in competitive athletics.^5,7^ Most patients with an asymptomatic pre-excitation will go through life without any related clinical events, but approximately one in five patients will develop an arrhythmia during follow-up and the first arrhythmic event may lead to sudden cardiac death (SCD).^5^ Exercise stress test (EST) has been widely used as a non-invasive tool for risk stratification especially in patients with asymptomatic pre-excitation. Sudden loss of pre-excitation during EST is accepted to be a low-risk predictor for malignant arrhythmias.^8^ However, some recent studies, which were mostly done in the paediatric population, showed that EST may have insufficient diagnostic accuracy for risk stratification in WPW patients.^9,10^ Despite the presence of limited and mostly retrospective data in adult WPW population, it seems that the risk of arrhythmic events is not homogenous in different age groups according to the previous reports.^9^ Prospective data analysing the adequacy of EST to rule out high-risk APs, particularly in the adult patient group, are still lacking.

This study aimed to clarify the usefulness of EST in risk stratification for adverse arrhythmic events in a large consecutive sample of adult patients who presented with asymptomatic or symptomatic pre-excitation and referred for risk assessment at a tertiary centre.

## Methods

### Study population

This prospective study included consecutive patients with documented ventricular pre-excitation, referred for assessment to the Arrhythmia Department at Karolinska University Hospital between August 2017 and December 2021. The study protocol was registered in the U.S. National Institutes of Health Clinical Trials Registry (ClinicalTrials.gov identifier: NCT03301935) and was approved by the Institutional Ethical Committee in Stockholm, DN:2016/1481-31/4 (2019-01205). Written informed consent was obtained from all patients.

All consecutive patients with ventricular pre-excitation documented by 12-lead ECG and without inability or contraindications to perform EST (bicycle) or EPS were asked to participate.

Enrolled patients were assigned to perform both an EST and an invasive EPS in that specific order. Crucial demographic, clinical, and EPS information were collected prospectively by treating electrophysiologist at the time of the EPS and stored in an electronic database containing all consecutive EPS at the Karolinska University Hospital (FileMaker Inc., Santa Clara, CA, USA). The ablation procedures were part of the clinical treatment to which the patients gave their informed consent.

### Exercise stress testing

An incremental EST was performed on a stationary cycle ergometer according to standard protocol. ECG was obtained before the EST and closely monitored during the EST to determine loss of pre-excitation. Patients with an absence of delta wave before the EST or with sudden loss of pre-excitation during the test were considered low risk. Patients with gradual loss of pre-excitation or persistent pre-excitation during exercise testing were defined as patients without low-risk EST parameters.

### Electrophysiological study

None of the patients were on antiarrhythmic medication before or during the study. Eight of 164 EPS were done under general anaesthesia. The remaining 156 procedures were performed with light sedation, under local anaesthesia, and in the fasting state. Heparin 5000 IU was given routinely after femoral vein puncture through a femoral sheath. A conventional EPS was performed with diagnostic catheters positioned in the right ventricular apex, in the coronary sinus, and in the His position. The EPS was performed according to a standard protocol. Antegrade and retrograde conduction properties of the AP were measured at baseline and during isoproterenol infusion. The conduction through the AP was considered rapid and possibly dangerous if either of the following conditions were met: APERP of ≤250 ms on a single atrial extra stimulus protocol. If APERP was >250 ms at baseline, atrial burst pacing down to the shortest cycle length with 1:1 activation of atria up to 30 s from two separate sites until AF was induced. The SPERRI ≤250 ms once again defined rapid conduction through the AP. The whole protocol was repeated during isoproterenol infusion unless APERP/SPERRI at baseline was ≤250 ms. The dose of isoproterenol was increased until the heart rate had increased to >50% from baseline or over 100 b.p.m. Any tachyarrhythmia induced during EPS was noted. The ablation procedures were part of the clinical treatment to which the patients gave their informed consent. Data from the EPS were gathered using the local EP registry and included APERP/SPERRI of the AP, inducibility and type of tachycardia, and AP localization.

### Statistical analysis

Numerical variables are expressed as mean ± standard deviation. Continuous variables were compared using Student’s *t*-test, and categorical variables were compared using the *χ*^2^ test or Fisher’s exact test. The sensitivity, specificity, positive predictive value (PPV), and negative predictive value (NPV) were calculated according to standard definitions. Diagnostic accuracy was defined as the proportion of subjects correctly classified by EST as having a low-risk AP upon EPS. A *P* < 0.05 was considered statistically significant. Statistical analyses were performed using SPSS version 25 software (IBM SPSS Inc., Chicago, IL, USA).

## Results

**Figure 1:**
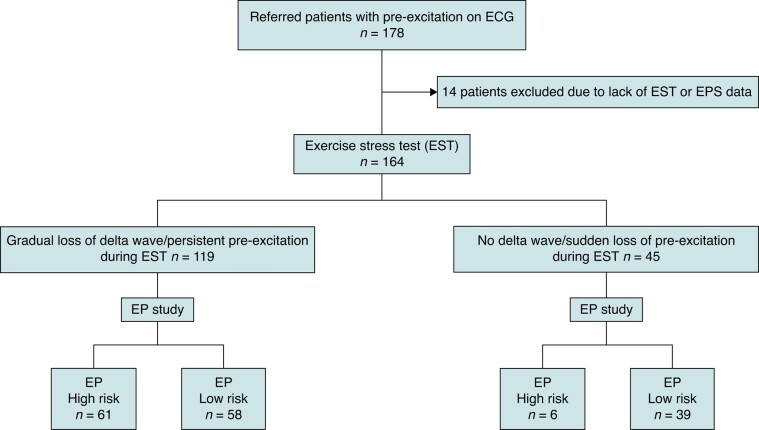
Electrophysiological high-risk: APERP/SPERRI ≤ 250 ms. APERP/SPERRI, accessory pathway effective refractory period/shortest pre-excited RR interval; EP study, electrophysiological study.

A total of 178 patients were included in the study (*Figure 1*). Fourteen patients were excluded due to the lack of either EST or EPS data. Baseline characteristics of the study group are shown in *Table 1*. The average age at the time for the EPS was 39 ± 17 years (range of 14–77). The majority (107, 64%) reported symptoms suggesting tachyarrhythmias before entering the study. The most common symptom was palpitation. Three patients had syncope, and one patient had experienced sudden cardiac arrest without any sequelae. Twenty-five patients had one or more types of documented tachycardia, of which orthodromic atrioventricular reciprocating tachycardia (AVRT) was the most common (*n* = 12; 7%). Five patients had documented pre-excited atrial fibrillation before enrolment.

**Table 1:** Baseline characteristics of study group, *n*: 168

Age mean (min–max)	39 (14–77)
Female, *n* (%)	66 (39)
Symptomatic, *n* (%)	107 (63)
Palpitation, *n*	107
Chest pain, *n*	1
Syncope, *n*	3
SCA, *n*	1
Documented arrhythmia, *n* (%)	25 (15)
Orthodromic AVRT	12 (7)
Antidromic AVRT	6 (3)
Atrial fibrillation	3 (2)
Pre-excited atrial fibrillation	5 (3)
HT, *n* (%)	17 (10%)
DM, *n* (%)	5 (3%)
ADHD, *n* (%)	8 (5%)

ADHD, attention deficit hyperactivity disorder; AVRT, atrioventricular reciprocating tachycardia; DM, diabetes mellitus; HT, hypertension; SCA, sudden cardiac death.

### Exercise stress testing

**Figure 2:**
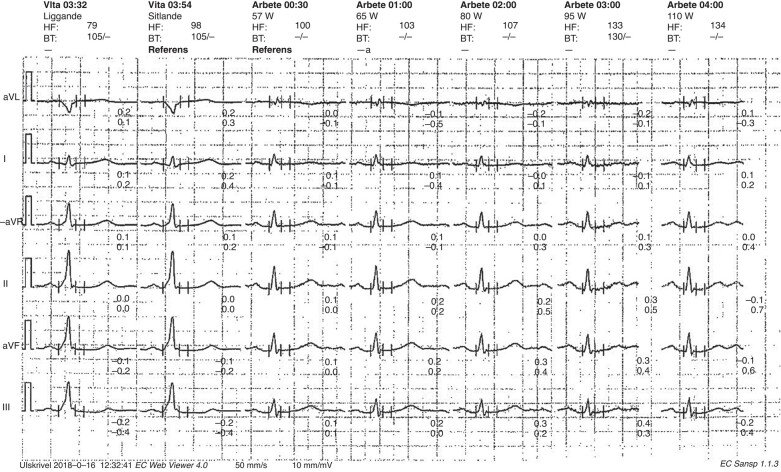
Sudden loss of pre-excitation during EST in an asymptomatic patient. EST, exercise stress test.

One hundred and sixty-four patients completed a conclusive EST. Forty-five patients (27%) demonstrated low-risk findings on EST, of which 19 were asymptomatic and 26 were symptomatic (*Figure 2* and *Table 2*). Four patients had narrow QRS complex tachycardia, and one patient had atrial fibrillation during EST.

**Table 2:** Non-invasive and invasive test characteristics of patients according to symptoms

	Asymptomatic, *n*: 61	Symptomatic, *n*: 107	*P*
Age (mean)	39 ± 15	40 ± 17	NS
Female, *n* (%)	21 (34)	45 (42)	NS
EST (*n* = 164)	59	105	
Low-risk parameters on EST^a^	19	26	NS
EP study (*n* = 164)	59	105	
Inducible arrhythmia, *n* (%)	36 (61)	73 (69)	NS
Orthodromic AVRT, *n*	15	56	
Antidromic AVRT, *n*	1	4	
Afib, *n*	15	19	
Pre-excited Afib, *n*	5	11	
AVNRT, *n*	–	5	
APERP mean (ms)	367 ± 135	351 ± 111	NS
SPERRI mean (ms)	335 ± 74	347 ± 64	NS
SPERRI/APERP ≤ 250 ms baseline, *n* (%)	7 (12)	10 (10)	
SPERRI/APERP ≤ 250 ms after Iso infusion, *n*: total (%)	24 (40)	43 (41)	NS
Ablation, *n* (%)	36 (61)	88 (83)	
General anaesthesia, *n*	8		
Complications			
Minor, *n*	4		
Inadvertent femoral artery puncture	3		
Temporary sinus node dysfunction	1		
Major, *n*	1		
Pericardial tamponade			

Afib, atrial fibrillation; APERP, accessory pathway effective refractory period; AVNRT, atrioventricular nodal re-entrant tachycardia; AVRT, atrioventricular re-entrant tachycardia; EP, electrophysiological study; EST, exercise stress test; NS, not significant; SPERRI, shortest pre-excited RR interval.

^a^Low-risk parameters: sudden loss of pre-excitation during test or no pre-excitation before/during EST.

### Electrophysiological features of accessory pathways in asymptomatic and symptomatic patients

An EPS was performed in 164 patients according to protocol (*Table 2*). Six of the 45 patients (13%) with low-risk EST had APERP/SPERRI ≤ 250 ms at EPS (*Figure 1*). The total number of asymptomatic subjects with low-risk EST was 19. Two patients in the asymptomatic group had APERP/SPERRI ≤ 250 ms despite showing low-risk findings at EST. The sensitivity, specificity, PPV, NPV, and accuracy of low-risk EST for excluding patients with SPERRI/APERP ≤ 250 ms were 40% (40), 91% (98), 87% (97), 51% (53), and 60% (64), respectively (the numbers in the parentheses indicate findings without isoproterenol, *Table 3*). The proportion of patients with APERP/SPERRI ≤ 250 ms was similar between the symptomatic and asymptomatic groups either with or without isoproterenol (symptomatic, 41%; asymptomatic, 40%; *P* = 0.23). Seventeen patients, 10 symptomatic and 7 asymptomatic, had APERP/SPERRI ≤250 ms on EPS without isoproterenol infusion. The number increased to 43 in symptomatic and 24 in asymptomatic group after isoproterenol infusion. The APERP/SPERRI could not be obtained in 14 patients; in 7/14 cases, no pathway could be demonstrated, and in 7 cases, the pathway was concealed under EPS. These patients were included in the analysis as patients without high-risk features.

**Table 3:** Relation of AP features with EST

	Low-risk EST^a^	Persistent pre-excitation during EST
APERP/SPERRI ≤ 250 ms, *n*	6	61
APERP/SPERRI > 250 ms, *n*	39	58

AP, accessory pathway; APERP, Accessory pathway effective refractory period; EST, exercise stress test; SPERRI, shortest pre-excited RR interval; Sensitivity: 40%; specificity: 91%; PPV: 87%; NPV: 51%; accuracy: 61%—of low-risk EST in excluding patients with SPERRI/APERP ≤ 250 ms.

^a^Definition of positive test result.

### Analysis of asymptomatic patients with low-risk exercise stress test parameters

The total number of asymptomatic subjects with low-risk EST parameters was 19. Two patients in the asymptomatic group had APERP/SPERRI ≤ 250 ms despite showing low-risk findings at EST. The sensitivity, specificity, PPV, NPV, and accuracy of low-risk EST for excluding patients with SPERRI/APERP ≤ 250 ms were 48, 92, 89, 55, and 66%, respectively (*Table 4*).

**Table 4:** Relation of AP features with EST in asymptomatic patients

	Low-risk EST^a^	Persistent pre-excitation during EST
APERP ≤ 250	2	22
APERP > 250	17	18

AP, accessory pathway; APERP, accessory pathway effective refractory period; EST, exercise stress test; Sensitivity: 48%; specificity: 92%; PPV: 89%; NPV: 55%; accuracy: 66%—of low-risk EST in excluding patients with SPERRI/APERP ≤ 250 ms.

^a^Definition of positive test result.

### Inducibility of arrhythmia and catheter ablation

The proportion of inducible arrhythmia was similar between asymptomatic and symptomatic patients (36 in asymptomatic patients, 61%; 73 in symptomatic patients, 69%; *P* = 0.28). The most common type of inducible tachycardia was orthodromic AVRT, which was induced in 71 (63%) cases (*Table 2*). Pre-excited atrial fibrillation was induced in 16 cases (9%). The number of patients with APERP ≤ 250 ms on EPS either with or without isoproterenol was significantly higher in patients with persistent pre-excitation compared to patients with low-risk EST (APERP ≤ 250 ms in 61 of 119 persistent pre-excitation; in 6 of 45 low-risk EST, *P* < 0.01, *Figure 1*). Ablation was performed in 88 (83%) symptomatic and 36 (61%) asymptomatic patients (*Table 2*). The most common localization for the AP was the left side (50%).

## Discussion

### Main findings

The main finding in this prospective study was that EST was insufficient to rule out patients with APs with high-risk properties regardless of symptoms. Furthermore, the proportion of rapid conducting pathways was similar in asymptomatic and symptomatic patients before or after isoproterenol administration.

### Risk stratification in asymptomatic pre-excitation

While patients with the WPW syndrome have been referred for invasive EPS and ablation on a routine basis for >25 years, this has not been the case for patients with asymptomatic pre-excitation. Previous studies including both symptomatic and asymptomatic patients found a similar risk of potentially life-threatening arrhythmias regardless of arrhythmia symptoms.^11,12^ The positive predictive value of an EPS regarding a potentially dangerous AP and the risk for serious adverse events have been considered too low to justify routine EPS and ablation in asymptomatic patients other than those with some high-risk occupations.^13^ This strategy, however, was based on small studies. The risk of SCD in asymptomatic pre-excitation is between 0.5 and 2 per 1000 patient-years.^14^ The risk of cardiac arrest/ventricular fibrillation in previously asymptomatic paediatric population has been estimated at 2.4 per 1000 person-years, but no deaths were reported in a registry study of 2169 patients over an 8-year follow-up period.^15^ It is not clear whether this result can be translated to the adult population, as children may underestimate the symptoms compared to adults. The present study included older population with mean age of 39 (range 14–77). The proportion of potentially dangerous APs and the mean APERP/SPERRI in symptomatic and asymptomatic groups were similar in our study. Arrhythmia was also induced in 61% of the asymptomatic patients compared to 69% in the symptomatic groups, suggesting that a large proportion of the asymptomatic patients either may have asymptomatic arrhythmia episodes or are prone to manifest clinical tachycardia in the future.

Since the study protocol was designed before the publication of the 2019 European Society of Cardiology (ESC) guidelines, symptomatic patients were also included in our study.^5^ We think that the value of EST in risk stratification is also important in symptomatic patients who refuse an invasive procedure or have limited access to EPS even though there is an indication for EPS in this group.^5^ In addition, due to the non-specific nature of arrhythmia symptoms, the symptom status may be challenging to interpret in some patients, which makes it difficult to identify truly asymptomatic patients.

### Exercise stress test as risk assessment

Sudden loss of pre-excitation during the EST has been accepted as a low-risk parameter previously.^8^ In our study, EPS revealed high-risk AP properties in 6 of 45 patients with intermittent or sudden loss of pre-excitation at EST (*Figure 1*). Non-invasive evaluation of the conducting properties of the AP in individuals with asymptomatic pre-excitation may be considered (Class IIb) according to the ESC guidelines.^5^ Exercise stress test is a common non-invasive test used for risk stratification in asymptomatic individuals with pre-excitation where identification of an abrupt and complete normalization of the PR interval with loss of delta wave during EST has been considered a marker of low risk.^8^ One study including 67 patients showed that continuous pre-excitation during an EST identified patients with WPW syndrome at risk for sudden death with a sensitivity of 80%, a specificity of 28.6%, and a predictive accuracy of 11.8%.^16^ A recent multi-centre analysis of paediatric population revealed that asymptomatic patients with non-persistent pre-excitation at EST can still present with high-risk of AP properties at EPS,^17^ in which 11% of patients with intermittent pre-excitation and high-risk AP at EPS were asymptomatic.^17^ Additionally, they found a low sensitivity (16%) and low NPV (23%) of non-persistent pre-excitation in excluding high-risk APs.^17^ We found a low sensitivity (40%) and low NPV (51%) of low-risk EST in the elimination of high-risk APs in both symptomatic and asymptomatic patients, which is in parallel with these results. When we analysed the asymptomatic patients separately, the sensitivity and NPV of low-risk EST in excluding high-risk APs increased to 48 and 55%, respectively. However, two asymptomatic patients with non-persistent pre-excitation at EST had APERP ≤ 250 ms on EPS, where the first symptom might be cardiac arrest. Our data show that a proportion of WPW patients with sudden loss of pre-excitation during EST may still have rapidly conducting AP and thus a risk for malignant arrhythmic events.

### Electrophysiological study for risk assessment

Variables in the EPS that identify patients with a high-risk AP include a SPERRI/APERP ≤ 250 ms, multiple APs, and an inducible AP-mediated tachycardia in the baseline state or during isoproterenol infusion. Our results showed no difference on two major variables indicating potentially high-risk AP, between symptomatic and asymptomatic patients. The proportion of potentially dangerous APs and the mean APERP/SPERRI value in symptomatic and asymptomatic groups were similar in our study. Obeyesekere and Klein^18^ suggest that invasive treatment in asymptomatic patients with WPW is indicated only for preventing SCD. It is claimed that the SCD risk is very low in asymptomatic WPW patients, as some of previous analyses found the SCD incidence 0% and others found 0.47% year/patient^12,19,20^ in the adult population, suggesting that ablation may cause more harm than benefit in real life.^18^ However, ablation is successful in ≥95% of cases today, the risk for major complications is considerably <1% of ablation attempts applying radiofrequency, and the risk related to an invasive EP study alone is far <1%.^21^ Our results suggest that all patients with pre-excitation should undergo EPS regardless of symptoms. Additionally, dyssynchronous mechanical contraction of the left ventricle is another concern in patients with pre-excitation. A recent study showed that children with asymptomatic pre-excitation had significantly lower VO_2_max and anaerobic thresholds compared to healthy controls.^22^ A case series revealed improvement in physical performance measurements and echocardiographic dyssynchrony parameters after catheter ablation in five patients with pre-excitation.^23^ However, further comprehensive studies are needed to assess the relation between myocardial performance and pre-excitation.

Our study showed that a considerable portion of asymptomatic patients had inducible arrhythmia (61%) and high-risk AP features (41%) during EPS. Twenty-two of 40 asymptomatic patients with persistent pre-excitation (55%) during EST had high-risk AP features and 14 of them (35%) had inducible AVRT in EP study. De Ponti *et al.*^24^ found that approximately half of asymptomatic patients with persistent pre-excitation during EST shows high-risk AP features and have inducible AVRT in EP study, which is consistent with our results. A number of studies demonstrated that one-fifth of patients with low-risk parameters on non-invasive evaluation have high-risk AP parameters on EP study.^10,25,26^ Some authors suggest that an invasive EPS is the unique method to uncover the antegrade conducting capability of APs in patients with pre-excitation.^11,15^ Absence or sudden loss of pre-excitation during non-invasive testing or during EST can be a sign of catecholamine sensitivity of AP conduction capability, which can limit the usefulness of non-invasive test in the assessment of AP antegrade conduction properties.^25^ Diagnostic EPS should involve isoproterenol infusion to assess the high-risk individuals according to the ESC guidelines.^5^ The number of patients with high-risk AP on EP study increased from one to six (13%) after isoproterenol infusion in the low-risk EST group in our study, which shows the crucial role of isoproterenol use in the assessment of antegrade AP conducting properties in the WPW population during EPS. The sensitivity and NPV of low-risk EST in eliminating high-risk APs became lower after isoproterenol use in EPS. Performing EPS under general anaesthesia has been shown to be independently associated with lower-risk AP properties.^17^ In this study, only eight EPS were performed under general anaesthesia, of which two represented patients with high-risk AP properties on EPS and, interestingly, both were in asymptomatic group.

Therefore, in terms of clinical implications, we consider our results to suggest that all patients with pre-excitation should undergo EPS regardless of symptoms.

### Limitations

A limitation of this study is the relatively small number of participants, which limits the statistical power. This is however, to the best of our knowledge, the largest prospective study done to evaluate the diagnostic value of EST to identify patients with a high-risk AP according to a consecutive invasive EPS.

Even though the rapid conduction capability of APs has been well correlated with the risk of future malignant arrhythmic events, procedural findings may not directly reflect the real-life SCD risk. Our findings were obtained in a single high-volume centre with a low rate of procedural complications, which may limit the application of our results to other settings.

## Conclusions

Sudden loss of pre-excitation during EST has a low sensitivity and a low negative predictive value to rule out potentially dangerous APs. Invasive EPS should be considered for risk evaluation in patients with pre-excitation regardless of symptoms. Routine isoproterenol use should be an integral part of EPS to overcome catecholamine sensitivity of APs and to prevent false negative results.

## Data Availability

The data supporting the findings of this study are available from the corresponding author upon reasonable request.
